# Supplementary material for: Actomyosin contractility-dependent matrix stretch and recoil induces rapid cell migration
Source: Nat Commun. 2019 Mar 12;10:1186. doi: 10.1038/s41467-019-09121-0 (PMC6414652; doi:10.1038/s41467-019-09121-0)
Supplement: Supplementary file 1 — Supplementary Information [file 41467_2019_9121_MOESM1_ESM.pdf]

Actomyosin contractility-dependent matrix stretch and recoil induces rapid cell migration

William Y. Wang[1], Christopher D. Davidson[1], Daphne Lin[1], and Brendon M. Baker[1*]

[1]Department of Biomedical Engineering, University of Michigan, Ann Arbor, MI 48109, USA

*Correspondence to: bambren@umich.edu

SUPPLEMENTARY MATERIAL

The Supplementary Material includes 11 Supplementary Figures and 8 Supplementary Movies

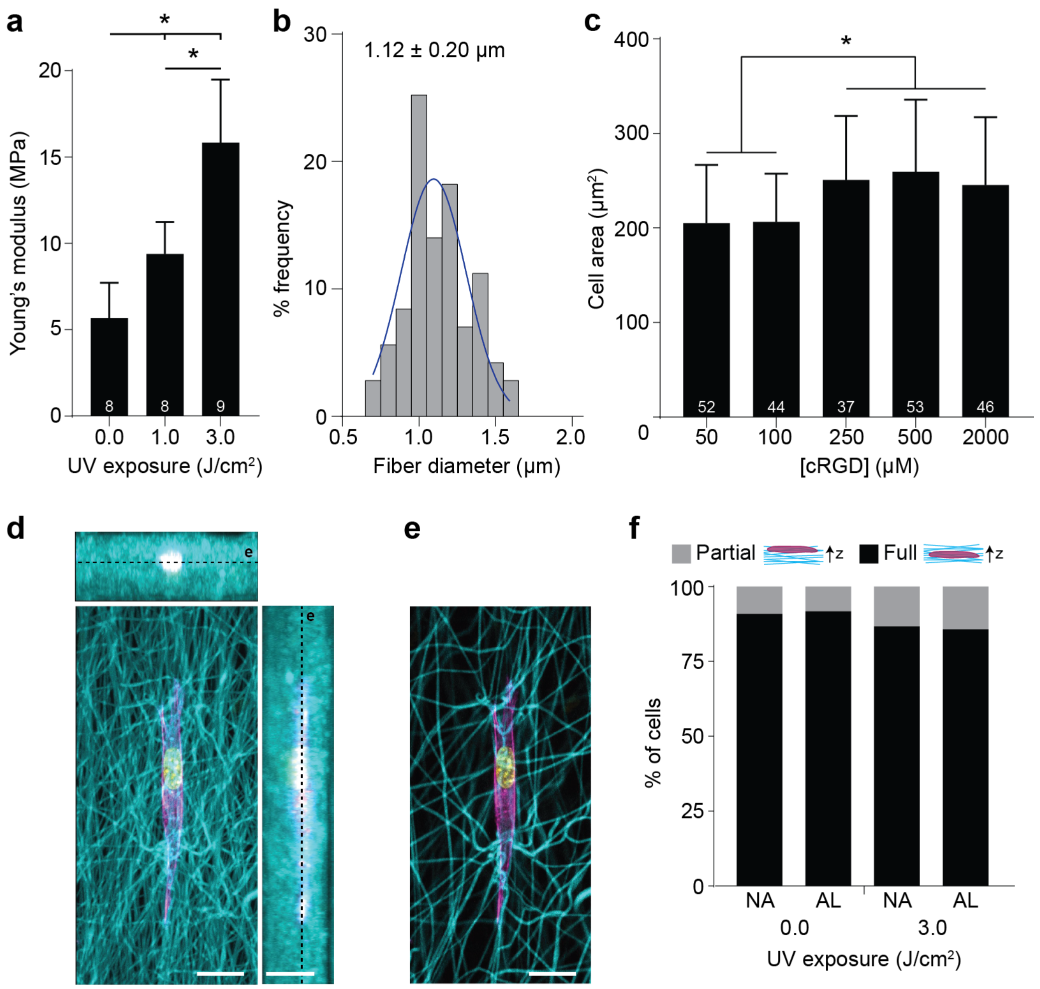

Supplementary Figure 1: Properties of DexMA fibrous matrices. (a) Young's modulus of individual fibers measured by AFM as a function of UV-initiated crosslinking. n=number of matrices analyzed per condition indicated within each bar. Data presented as mean ± s.d. (b) Histogram of DexMA fiber diameter resulting from 47.5 wt% polymer solution (n=71 fibers). (c) NIH3T3 spread area as a function of [cRGD]. 500 µM cRGD was utilized for all migration studies unless otherwise noted. n=number of cells analyzed per condition indicated within each bar. Data presented as mean ± s.d. (d-e) Orthogonal x-y, x-z, and y-z maximum intensity projections (d) and single x-y plane (e) views of a composite confocal fluorescence image of representative NIH3T3 fibroblast within a suspended 3D fibrous matrix (rhodamine-labeled matrix fibers (cyan), F-actin (magenta), and nuclei (yellow); scale bars: 20 µm). (f) Percentage of cells that are partially or fully embedded within suspended fiber matrices 6 hours post seeding (n=30 cells per condition). * indicates a statistically significant comparison with $p<0.05$ (one-way ANOVA).

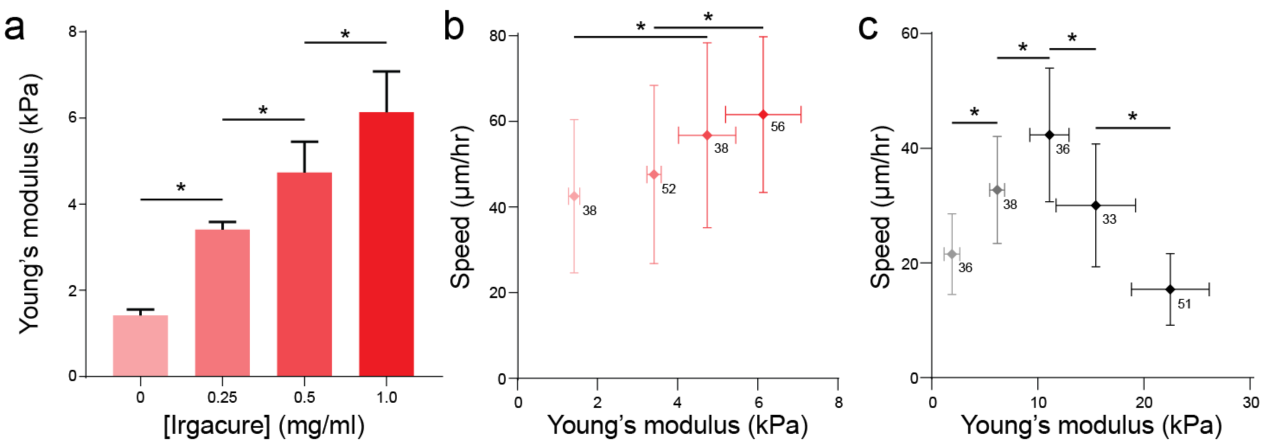

Supplementary Figure 2: Bulk mechanical properties influence cell migration speeds. (a) Bulk mechanical testing of aligned matrices as a function of Irgacure photoinitiator (I2959) concentration with a constant UV exposure of 1 J cm^{-2} (n=6 matrices per group). (b) NIH3T3 fibroblast migration speed as a function of Young's modulus in aligned matrices tuned by I2959 concentrations (a). n=number of cells per group as indicated beside each data point. (c) Human foreskin fibroblast migration speed as a function of Young's modulus in aligned matrices with a constant 1.0 mg ml^{-1} I2959, but varying durations of UV exposure (as in Figure 1d). n=number of cells per group as indicated beside each data point. All data presented as mean ± s.d. * indicates a statistically significant comparison with $p<0.05$ (one-way ANOVA).

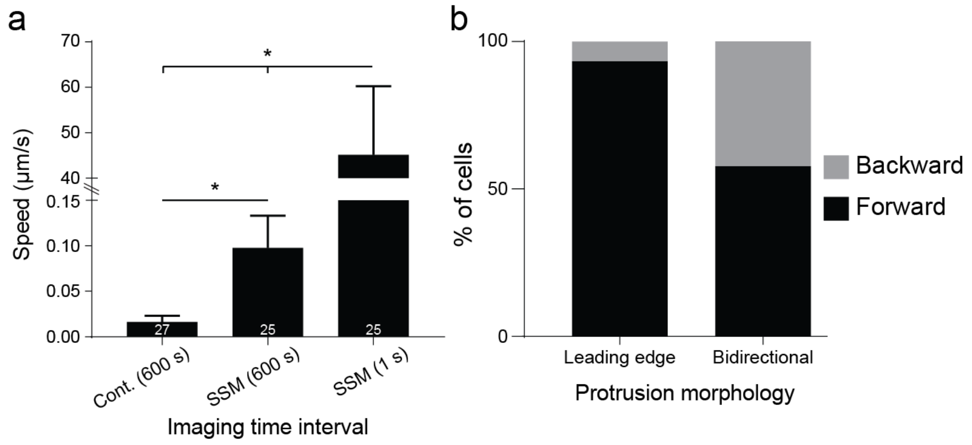

Supplementary Figure 3: Additional characteristics of slingshot migration. (a) Capturing matrix recoil events under transmitted light at 1 second frame intervals (SSM (1 s)) reveals more accurate matrix recoil speeds when compared to 10-minute frame intervals (SSM (600 s)).

n=number of cells per group as indicated within each bar. Data presented as mean ± s.d. (b) Percentage of cells employing SSM that recoil in the same direction (forward) or in the reverse direction (backward) with respect to its direction of continuous migration prior to SSM. Cells were parsed into two categories (leading edge and bidirectional) based on the morphology of cell protrusions during matrix stretch (n=30 cells per group). * indicates a statistically significant comparison with $p<0.05$ (one-way ANOVA).

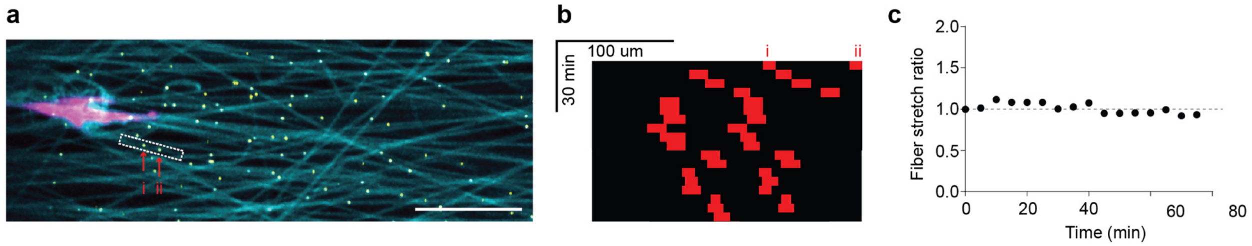

Supplementary Figure 4: Non-engaged fibers do not undergo stretch. (a) Composite confocal fluorescence image of an NIH3T3 in an intermediate stiffness matrix (matrix fibers (cyan), cytoplasm (magenta), and fiber-embedded beads (yellow); scale bar: 50 μm). (b) Kymograph of a pair of microspheres embedded within the same fiber indicated by red arrows in (a) that is not directly engaged by the cell. (c) Determination of fiber stretch ratio (relative to initial distance between fiducial markers) as a function of time.

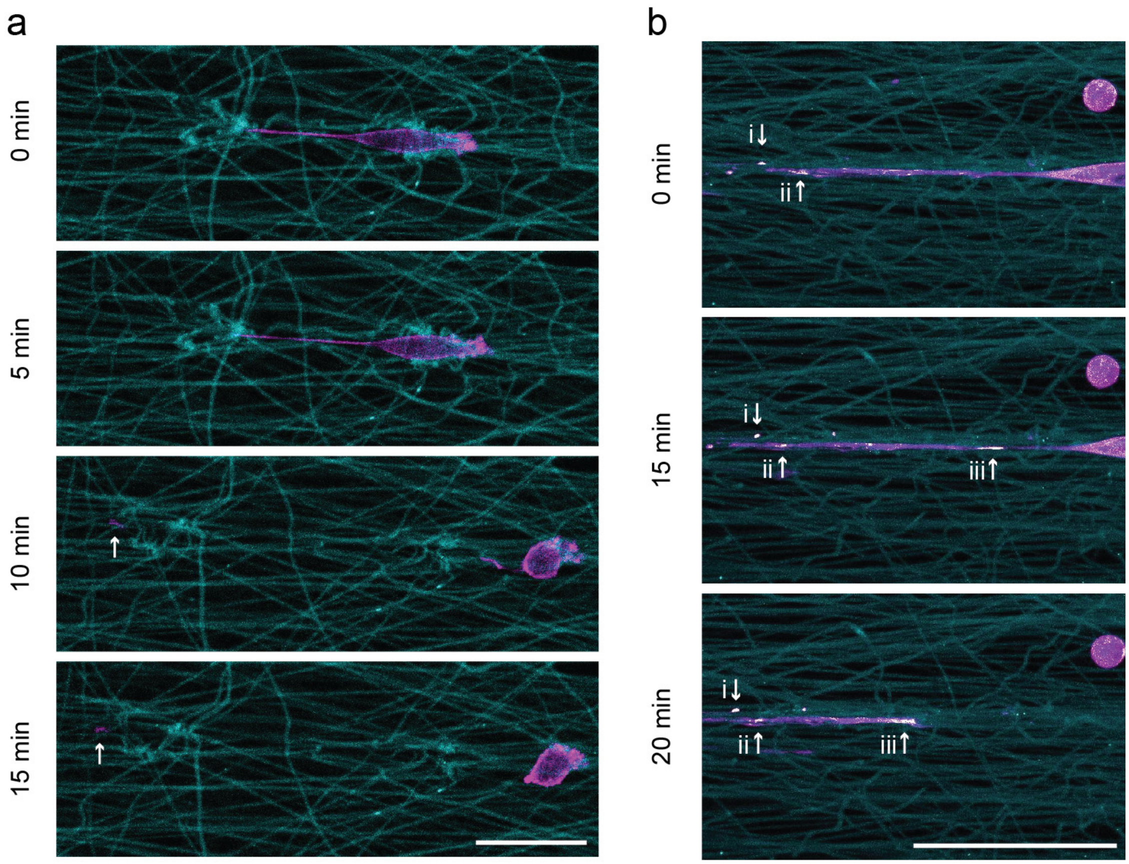

Supplementary Figure 5: Rupture of trailing edge concurrent with matrix recoil. (a) Select frames from confocal fluorescence time-lapse imaging shown in Supplemental Movie 5 of Lifeact-GFP expressing NIH3T3s within an aligned, intermediate stiffness matrix (matrix fibers (cyan), Lifeact-GFP (magenta); scale bar: 50 µm). Arrows indicate Lifeact-GFP puncta tethered to the matrix with matrix recoil. (b) Select frames from confocal fluorescence time-lapse imaging shown in Supplemental Movie 6 of Paxillin-GFP expressing NIH3T3s within an aligned, intermediate stiffness matrix (matrix fibers (cyan), cytoplasm (magenta), and paxillin (white), scale bar: 50 µm. Arrows highlight paxillin-rich puncta that remain tethered to the matrix within actin containing pieces of the cytoplasm that are separated from the cell upon matrix recoil. Roman numerals indicate paxillin-rich puncta associated across time frames.

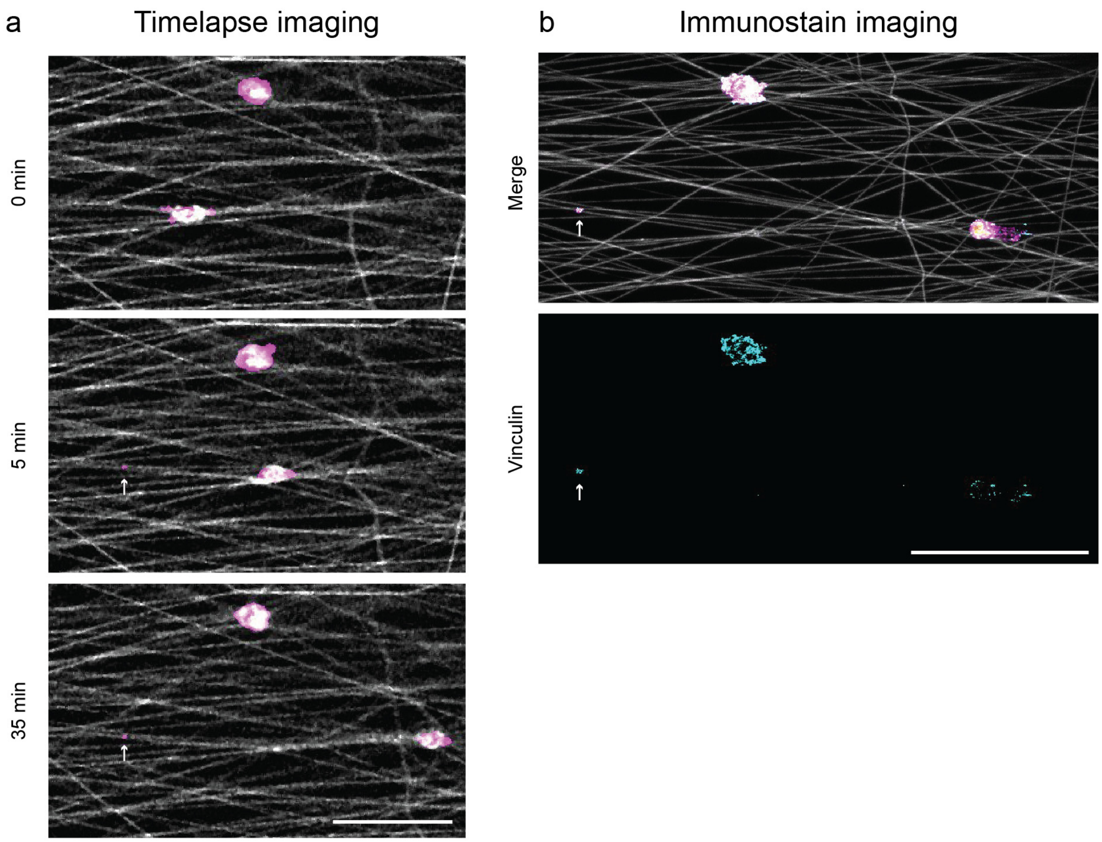

Supplementary Figure 6: Confirmation of vinculin within ruptured trailing edge plaques that contain paxillin. (a) Select frames from confocal fluorescence time-lapse imaging of NIH3T3-Lifeact-GFP cells within an aligned, intermediate stiffness matrix (matrix fibers (gray), Lifeact-GFP (magenta), and nuclei (yellow); scale bar: 50 µm). Matrix deformations are less visible due to low resolution imaging required for the sake of throughput. Arrow indicates Lifeact-GFP puncta tethered to matrix following matrix recoil. (b) Confocal fluorescence images of identical location as in (a), subsequently immunostained for vinculin directly on the microscope stage (matrix fibers (gray), Lifeact-GFP (magenta), nuclei (yellow), and vinculin (cyan); scale bar: 50 µm).

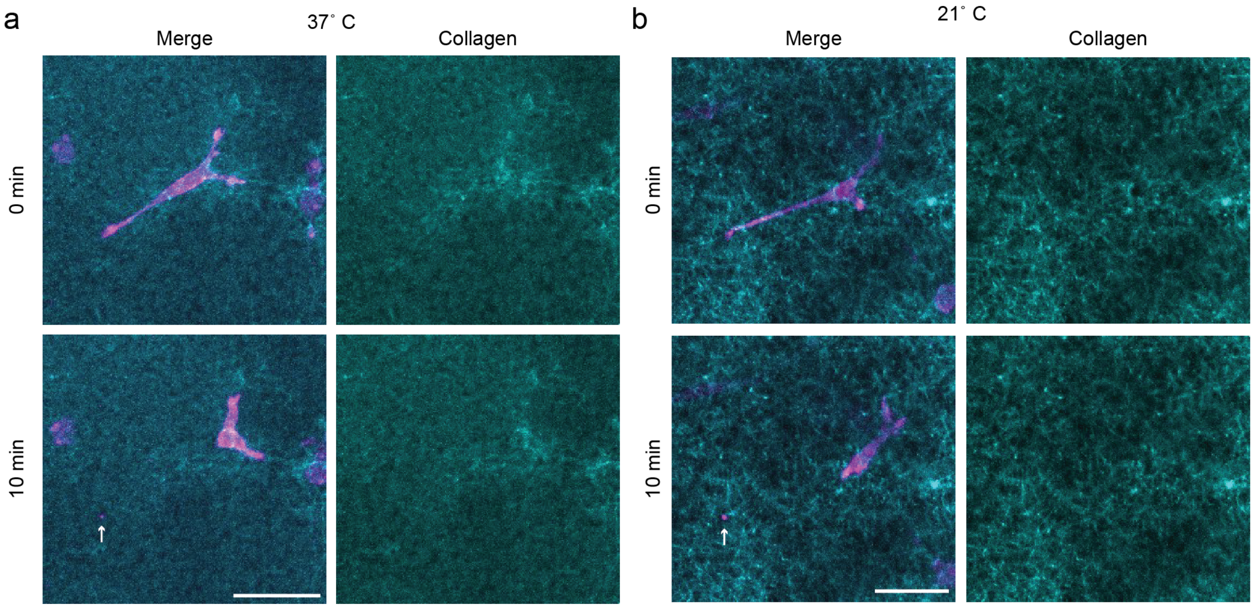

Supplementary Figure 7: Slingshot migration within 3D type I collagen hydrogels. (a-b) Select frames from confocal fluorescence time-lapse imaging (Supplemental Movie 7) of embedded NIH3T3-Lifeact-GFP cell migrating within Alexa555-succidinyl ester labeled 1.0 mg ml^{-1} collagen 3D hydrogels (collagen-Alexa555 (cyan), Lifeact-GFP (magenta); scale bars: 50 µm). Collagen gels were formed at 37° C (a) and 21° C (b). Arrows indicate Lifeact-GFP puncta left behind in the collagen following matrix recoil and slingshot migration.

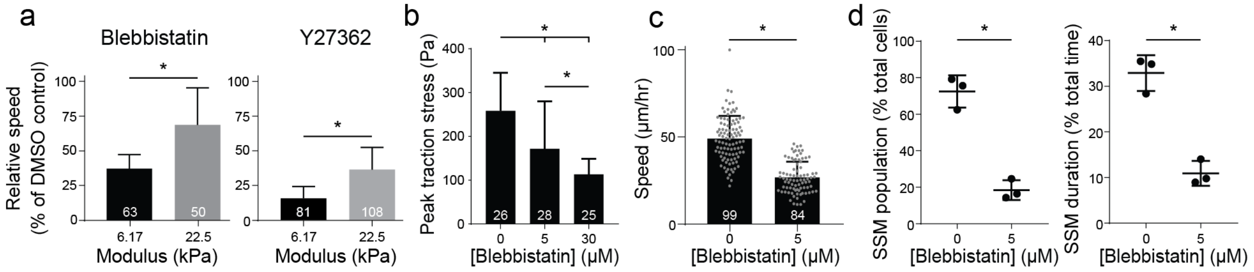

Supplementary Figure 8: Influence of contractility perturbations on slingshot migration. (a) Relative migration speed (normalized to DMSO control for each matrix stiffness) upon treatment with 30 µM blebbistatin or 25 µM Y27362 treatment. (b) Peak traction stress of NIH3T3s as a function of blebbistatin concentration measured via traction force microscopy on 7.9 kPa PAAm hydrogels. (c-d) Migration speed, SSM population (quantified over a 6-hour duration) and SSM duration of NIH3T3s treated with an intermediate blebbistatin dosage (5 µM) on aligned, intermediate stiffness matrices. n=3 fields of view; field of view=10 cells (d). All data presented

as mean ± s.d. * indicates a statistically significant comparison with p<0.05 (a, c-d: two-sided student's t-test; b: one-way ANOVA). For (a-c), n=number of cells per group as indicated within each bar.

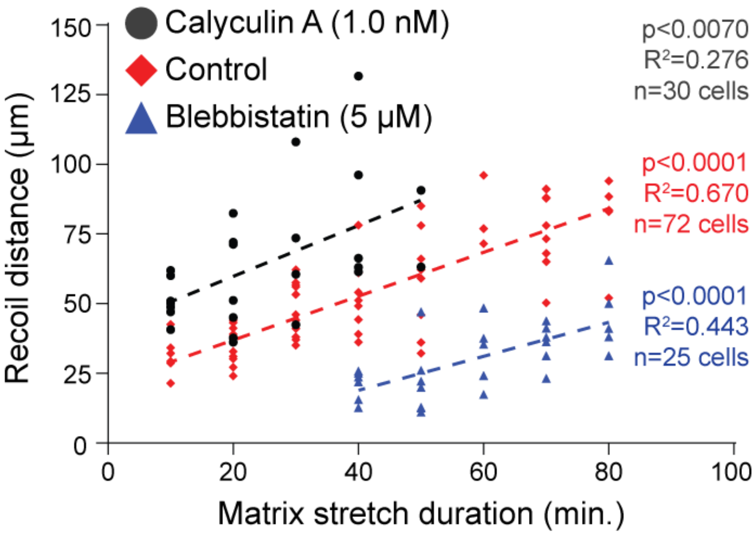

Supplementary Figure 9: Matrix stretch duration and recoil distance is dependent on intracellular contractility. Recoil distance (net translocation of cell) as a function of duration spent stretching the matrix. Dashed lines indicate corresponding linear correlations with indicated R^2 and p-values (linear regression). n=number of cells per group indicated within the plot.

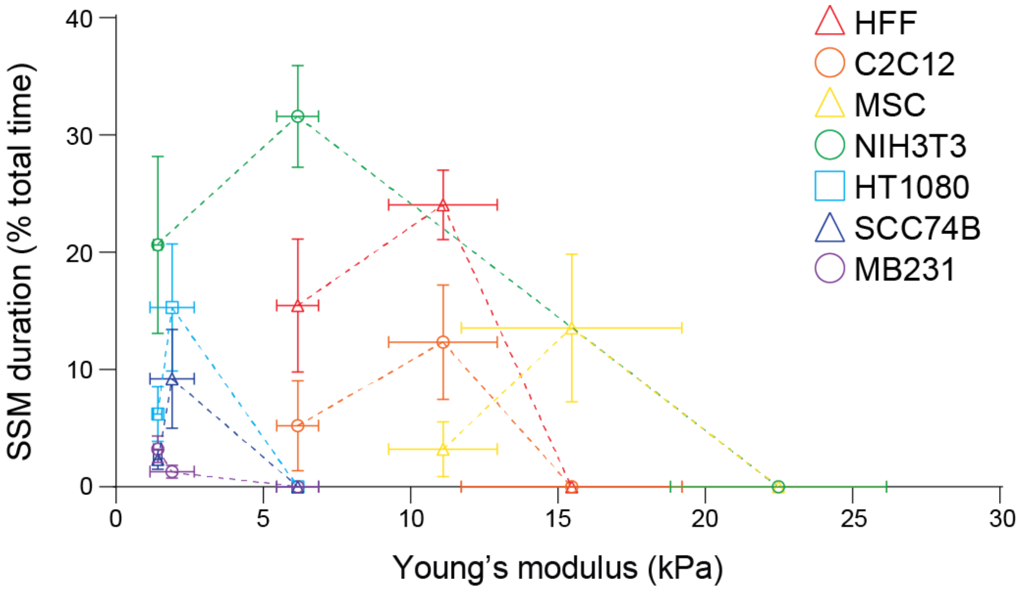

Supplementary Figure 10: Optimal stiffness for slingshot migration varies by cell type. Percentage of total imaging duration cells spent in some phase of matrix stretch and recoil (SSM duration) as a function of Young's modulus for a variety of cell types (n=3 fields of view per group; field of view=10 cells). Dashed lines connect data points for each cell type. Data presented as ± s.d.

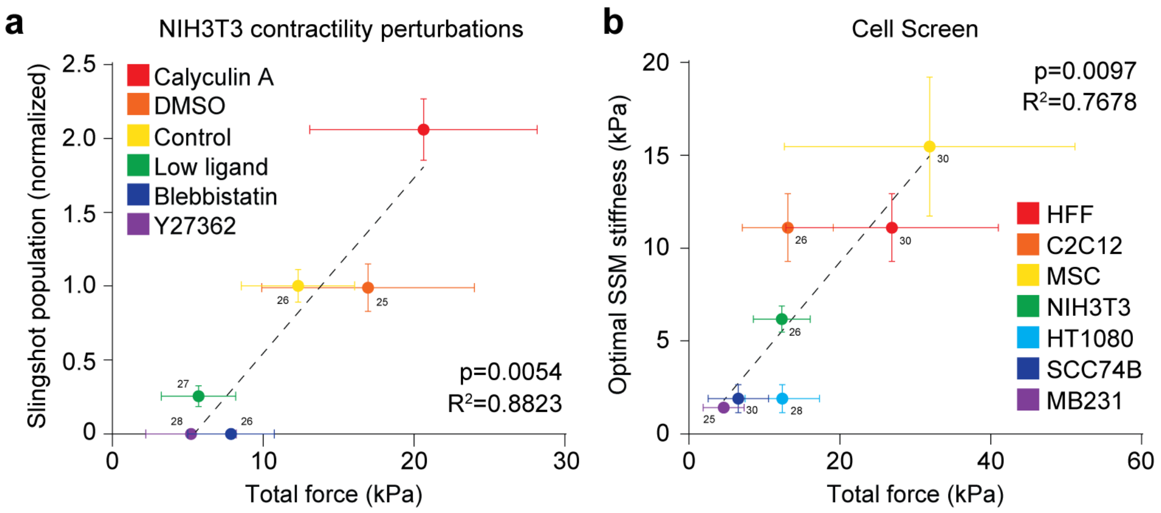

Supplementary Figure 11: SSM population and optimal SSM stiffness scales with total traction forces. (a) NIH3T3 slingshot population on aligned, intermediate stiffness matrices (normalized to DMSO control) positively correlates with total contractile force determined by traction force microscopy. Dashed line indicates linear correlation with indicated R^2 and p-value=0.0054 (linear regression). n=number of cells per group as indicated beside each data point. (b) Matrix stiffness corresponding to optimal slingshot migration positively correlates with total contractile force for a variety of cell types. Dashed line indicates linear correlation with indicated R^2 and p-value=0.0097 (linear regression). n=number of cells per group as indicated beside each data point. All data presented as mean ± s.d.
