# Supplementary material for: Actomyosin contractility-dependent matrix stretch and recoil induces rapid cell migration
Source: Nat Commun. 2019 Mar 12;10:1186. doi: 10.1038/s41467-019-09121-0 (PMC6414652; doi:10.1038/s41467-019-09121-0)
Supplement: Supplementary file 3 — Description of Additional Supplementary Files [file 41467_2019_9121_MOESM3_ESM.pdf]

Description of Additional Supplementary Files

File Name: Supplementary Movie 1
Description: Representative confocal fluorescence time-lapse imaging (10x magnification) of NIH3T3 migration within aligned and non-aligned matrices of low, intermediate, and high stiffness (movies play successively and are labeled individually; matrix fibers (cyan), cytoplasm (magenta), nuclei (yellow); scale bar: 100 μm).

File Name: Supplementary Movie 2
Description: Representative confocal fluorescence time-lapse imaging (32x magnification) of NIH3T3 migration within aligned matrices of low, intermediate, and high stiffness undergoing continuous migration and SSM within aligned matrices of intermediate stiffness (movies play successively and are labeled individually; matrix fibers (cyan), cytoplasm (magenta), nuclei (yellow); scale bar: 50 μm).

File Name: Supplementary Movie 3
Description: High temporal resolution transmitted light time lapse imaging (5x magnification) of NIH3T3 migration undergoing matrix recoil within aligned matrix of intermediate stiffness (scale bar: 50 μm).

File Name: Supplementary Movie 4
Description: Representative confocal fluorescence time-lapse imaging (32x magnification) of NIH3T3 matrix deformations during migration within aligned matrices of low, intermediate, and high stiffness matrices (movies play successively and are labeled individually; matrix fibers (cyan), cytoplasm (magenta), fiber-embedded fluorescent microspheres (yellow); scale bar: 50 μm).

File Name: Supplementary Movie 5
Description: Representative confocal fluorescence time-lapse imaging (32x magnification) of NIH3T3-LA-GFP migration within aligned, intermediate stiffness matrices (matrix fibers (cyan) and Lifeact-GFP (magenta); scale bar: 50 μm). Track marked in yellow highlights Lifeact-GFP puncta separated from the cell's trailing edge that remains tethered to the recoiled matrix.

File Name: Supplementary Movie 6
Description: Representative confocal fluorescence time-lapse imaging (32x magnification) of NIH3T3-Paxillin-GFP migration within aligned, intermediate stiffness matrices (matrix fibers (cyan), cytoplasm (magenta), and paxillin (white); scale bar: 50 μm). Marked track denotes paxillin-containing focal adhesion.

File Name: Supplementary Movie 7
Description: Representative confocal fluorescence time-lapse imaging (10x magnification) of embedded NIH3T3-LA-GFP within fluorescently labelled 1.0 mg ml-1 type I collagen hydrogels crosslinked at 37° C and 21° C (collagen-Alexa555 (cyan), and Lifeact-GFP (magenta); scale bar: 50 μm). Arrows denote Lifeact-GFP puncta separated from cells within the collagen matrix.

File Name: Supplementary Movie 8
Description: Representative transmitted light and fluorescence time-lapse imaging (10x magnification) of NIH3T3 migration within aligned, intermediate stiffness matrices treated with

DMSO, 30μM blebbistatin, or 1.0 nM calyculin A. Note: transmitted light was utilized for blebbistatin studies to avoid photoinactivity by blue light[1] . Scale bar: 100 μm
