# Supplementary material for: Actomyosin contractility-dependent matrix stretch and recoil induces rapid cell migration
Source: Nat Commun. 2019 Mar 12;10:1186. doi: 10.1038/s41467-019-09121-0 (PMC6414652; doi:10.1038/s41467-019-09121-0)
Supplement: Supplementary file 12 — Reporting Summary [file 41467_2019_9121_MOESM12_ESM.pdf]

nature research

Corresponding author(s): NCOMMS-18-06263B

Last updated by author(s): Jan 17, 2019

Reporting Summary

Nature Research wishes to improve the reproducibility of the work that we publish. This form provides structure for consistency and transparency in reporting. For further information on Nature Research policies, see Authors & Referees and the Editorial Policy Checklist.

Statistics

For all statistical analyses, confirm that the following items are present in the figure legend, table legend, main text, or Methods section.

n/a	Confirmed	
☐	☒	The exact sample size (*n*) for each experimental group/condition, given as a discrete number and unit of measurement
☐	☒	A statement on whether measurements were taken from distinct samples or whether the same sample was measured repeatedly
☐	☒	The statistical test(s) used AND whether they are one- or two-sided<br>*Only common tests should be described solely by name; describe more complex techniques in the Methods section.*
☐	☒	A description of all covariates tested
☐	☒	A description of any assumptions or corrections, such as tests of normality and adjustment for multiple comparisons
☐	☒	A full description of the statistical parameters including central tendency (e.g. means) or other basic estimates (e.g. regression coefficient) AND variation (e.g. standard deviation) or associated estimates of uncertainty (e.g. confidence intervals)
☐	☒	For null hypothesis testing, the test statistic (e.g. *F*, *t*, *r*) with confidence intervals, effect sizes, degrees of freedom and *P* value noted<br>*Give P values as exact values whenever suitable.*
☒	☐	For Bayesian analysis, information on the choice of priors and Markov chain Monte Carlo settings
☒	☐	For hierarchical and complex designs, identification of the appropriate level for tests and full reporting of outcomes
☒	☐	Estimates of effect sizes (e.g. Cohen's *d*, Pearson's *r*), indicating how they were calculated

Our web collection on statistics for biologists contains articles on many of the points above.

Software and code

Policy information about availability of computer code

Data collection	The ZEN microscopy software platform was used for the acquisition of all microscopy data in this manuscript.
Data analysis	Custom MATLAB code used for data analysis can be downloaded from DOI 10.6084/m9.figshare.7591205

For manuscripts utilizing custom algorithms or software that are central to the research but not yet described in published literature, software must be made available to editors/reviewers. We strongly encourage code deposition in a community repository (e.g. GitHub). See the Nature Research guidelines for submitting code & software for further information.

Data

Policy information about availability of data

All manuscripts must include a data availability statement. This statement should provide the following information, where applicable:

- Accession codes, unique identifiers, or web links for publicly available datasets
- A list of figures that have associated raw data
- A description of any restrictions on data availability

The data that support the findings of this study are available from the corresponding author upon reasonable request.

Field-specific reporting

Please select the one below that is the best fit for your research. If you are not sure, read the appropriate sections before making your selection.

☒ Life sciences ☐ Behavioural & social sciences ☐ Ecological, evolutionary & environmental sciences

For a reference copy of the document with all sections, see nature.com/documents/nr-reporting-summary-flat.pdf

Life sciences study design

All studies must disclose on these points even when the disclosure is negative.

Sample size	Sample size was not predetermined by statistical estimates; sample size was approximated based on standards from the field established in previous publications from other groups
Data exclusions	Exclusion criteria was established a priori - namely all cells that did not spread or migrate during the window of observation were excluded form the analysis, which is a field standard.
Replication	All experiments including quantification of migration speed were replicated three times.
Randomization	Not relevant - no randomization was required for the experimental design of any of these experiments.
Blinding	Not relevant - no blinding was required for the experimental design of any of these experiments, as none of the assessments were subjective.

Reporting for specific materials, systems and methods

We require information from authors about some types of materials, experimental systems and methods used in many studies. Here, indicate whether each material, system or method listed is relevant to your study. If you are not sure if a list item applies to your research, read the appropriate section before selecting a response.

Materials & experimental systems

n/a	Involved in the study	
☐	☒	Antibodies
☐	☒	Eukaryotic cell lines
☒	☐	Palaeontology
☒	☐	Animals and other organisms
☒	☐	Human research participants
☒	☐	Clinical data

Methods

n/a	Involved in the study	
☒	☐	ChIP-seq
☒	☐	Flow cytometry
☒	☐	MRI-based neuroimaging

Antibodies

Antibodies used	Anti-vinculin mouse monoclonal, hVIN-1, Sigma-Aldrich, V9264, 047M4795V
Validation	Validated by manufacturer via immunoblotting

Eukaryotic cell lines

Policy information about cell lines

Cell line source(s)	NIH3T3 fibroblasts (ATCC CRL-1658), human foreskin fibroblasts (Kenneth Yamada, NIH), human mesenchymal stem cells (Lonza PT-2501) , C2C12 (ATCC CRL-1772), UM-SCC-74B (Thomas Carey, University of Michigan), HT1080 (ATCC CCL-121), MDA-MB-231 (ATCC HTB-26)
Authentication	Cell lines were authenticated based on established morphology
Mycoplasma contamination	All cell lines used in these studies were tested for mycoplasma and confirmed to be negative.
Commonly misidentified lines (See ICLAC register)	N/A
